# RhoBTB3 Functions as a Novel Regulator of Autophagy by Suppressing AMBRA1 Stability

**DOI:** 10.3390/cells13191659

**Published:** 2024-10-07

**Authors:** Kyungho Kim, Dong-Gun Kim, Youn-Jae Kim

**Affiliations:** Targeted Therapy Branch, Division of Rare and Refractory Cancer, Research Institute, National Cancer Center, Goyang 10408, Republic of Korea

**Keywords:** AMBRA1, autophagy, RhoBTB3

## Abstract

Autophagy is essential for cell survival and cellular homeostasis under various stress conditions. Therefore, autophagy dysfunction is associated with the pathogenesis of various human diseases. We explored the regulatory role of RhoBTB3 in autophagy and its interaction with activating molecules in AMBRA1. RhoBTB3 deficiency was found to induce autophagy, while its overexpression inhibited autophagy induction. Through immunoprecipitation and mass spectrometry, AMBRA1 was identified as a substrate of RhoBTB3. The study revealed that RhoBTB3 regulates AMBRA1 stability by influencing its protein levels without affecting its mRNA levels. RhoBTB3 induced the ubiquitination of AMBRA1, leading to proteasome-mediated degradation, with the ubiquitination occurring at K45 on AMBRA1 through a K27-linked ubiquitin chain. The knockdown of AMBRA1 blocked RhoBTB3 knockdown-induced autophagy, indicating the dependency of autophagy on AMBRA1. Thus, RhoBTB3 negatively regulates autophagy by mediating AMBRA1 ubiquitination and degradation, suggesting RhoBTB3 as a potential therapeutic target for autophagy-related diseases.

## 1. Introduction

Autophagy is an essential cellular degradation process in eukaryotes that promotes the recycling and reuse of damaged organelles and cell compartments [1,2]. It plays a pivotal role in maintaining cell homeostasis, participating in pathophysiological processes, influencing aging, and contributing to various diseases such as cancer, neurodegeneration, and autoimmune diseases [3,4]. Autophagy is initiated by the nucleation of a membrane vesicle called the phagophore, which extends and folds onto itself to create an autophagosome and is subsequently transported to lysosomes for degradation and recycling [5,6].

The initiation of autophagy is prompted by various factors such as nutrient deprivation, stress, and pathogen infections [7,8]. Under starvation stimulation, the inactivation of mTOR and the subsequent activation of Unc-51-like autophagy-activating kinases 1(ULK1) leads to autophagy induction [5,9]. The autophagosome formation is mainly controlled by the Beclin1- Phosphatidylinositol 3-kinase catalytic subunit type 3 (PIK3C3) complex [10,11]. ULK1 phosphorylates Beclin1, facilitating its binding to PIK3C3 [12,13]. This phosphorylation process results in the generation of phosphatidylinositol 3-phosphate (PI3P), a crucial element necessary for the formation of the autophagosome structure [14,15].

Activating molecule in Beclin1-regulated autophagy protein 1 (AMBRA1) has been reported to modulate the Beclin1-PIK3C3 complex which is crucial for the initiation of autophagy [16,17,18]. This process subsequently strengthens the interaction between Beclin1 and PIK3C3, promoting the assembly of autophagosomes [10]. AMBRA1 has been reported to be regulated by various post-translational modifications, such as phosphorylation and ubiquitination [19,20,21].

The Rho-related Broad-complex, Tramtrack, and Bric-à-brac (RhoBTB) subfamily comprises three members: RhoBTB1, RhoBTB2, and RhoBTB3, which is the most divergent isoform [22,23,24]. RhoBTB3 has been identified as an adapter protein for the E3 ubiquitin ligase, interacting with various target substrates, thereby influencing various cellular processes such as the cell cycle, collagen synthesis, and Warburg effect [25,26,27,28,29]. However, the role of RhoBTB3 in the regulation of autophagy remains underexplored. In this study, we aim to investigate whether RhoBTB3 is related to autophagy and explore its underlying mechanisms.

## 2. Materials and Methods

### 2.1. Plasmids and siRNA

To generate FLAG-RhoBTB3, human RhoBTB3 cDNAs were generated by the reverse transcription of RNAs from 293T cells using the SuperScript™ III First-Strand Synthesis System (#18080051, Thermo Scientific, Cleveland, OH, USA), and the cDNA was PCR amplified using the forward primer 5′-CAAGCGGCGCGCCATGTCCATCCAC-3′ and the reverse primer 5′-TAGAGGCTCGAGTTAATTAATTAC-3′. The amplified product was inserted into the pCS2-FLAG vector between the AscI and XhoI sites. pLPCX-Myc-AMBRA1 was provided by Francesco Cecconi (Tor Vergata University of Rome) [20]. The following human LC3 and human ubiquitin (Ub) plasmids were purchased from Addgene (Cambridge, MA, USA): ptfLC3 (mRFP-EGFP-LC3; # 21074), pRK5-HA-Ub-WT (#17608), pRK5-HA-Ub-K0 (#17603), pRK5-HA-Ub-K6 (#22900), pRK5-HA-Ub-K11 (#22901), pRK5-HA-Ub-K27 (#22902), pRK5-HA-Ub-K29 (#22903), pRK5-HA-Ub-K33 (#17607), pRK5-HA-Ub-K48 (#17605), and pRK5-HA-Ub-K63 (#17606) and pRK5-HA-Ub-K27R (#121155). The siRNAs used to target human RhoBTB3 (#1: 5′-CUGUGUUAG UACAACUGAA-3′, #2: 5′-CUGACAUCAUUGUGAUCAA-3′) and human AMBRA1 (#1: 5′-GUGAUGACGAACCAGAGAU-3′, #2: 5′-CACACUGUGGGC UUAA UGU-3′) and the non-targeting siRNA control (5′-CCUACGCCACCAAUUUCGU-3′) were all purchased from Bioneer (Daejeon, Republic of Korea).

### 2.2. Antibodies and Reagents

To determine the protein expression levels, we performed immunoblotting using the following antibodies: rabbit anti-AMBRA1 (#A302-568A, 1:1000, Bethyl Laboratories, Montgomery, TX, USA), anti-mouse FK2 anti-Ub (#BML-PW8810, 1:1000, Enzo Life Sciences, Farmingdale, NY, USA), anti-Myc (#6286-1-AP, 60003-2-Ig, 1:2000, Proteintech, Rosemont, IL, USA), anti-RhoBTB3 (13945-1-AP, Proteintech), anti-LC3B (#L7543, 1:3000, Sigma-Aldrich, St. Louis, MO, USA), anti-FLAG (F3165, F7425, 1:1500, Sigma-Aldrich), anti-HA-Tag (#3724, 1:2000, Cell Signaling, Beverly, MA, USA), and β-Actin (#5125, 1:3000, Cell Signaling). The secondary antibodies used are anti-rabbit-HRP (1706515, Bio-Rad, Hercules, CA, USA) and anti-mouse-HRP (1706516, Bio-Rad).

Cycloheximide (CHX; C4859), dimethyl sulfoxide (DMSO; D4540), and 1, 4-Dithiothreitol (DTT; D0632) were purchased from Sigma-Aldrich. MG132 (474790) was purchased from Calbiochem (La Jolla, CA, USA).

### 2.3. Cell Culture and Transfection

The human cervical carcinoma (HeLa), human lung cancer (A549), and human embryonic kidney (293T) cell lines were purchased from the American Type Culture Collection (ATCC; Manassas, VA, USA). The cells were maintained in high-glucose Dulbecco’s modified Eagle medium (DMEM) with 10% fetal bovine serum (FBS) and 1% penicillin–streptomycin. The cells were maintained in a water-jacketed incubator at 37 °C with 5% CO_2_ enrichment. For autophagy induction, the HeLa and A549 cells were treated with Hanks’ Balanced Salt Solution (HBSS; LB 003-02, Welgene, Gyeongsan, Republic of Korea) to induce stimulation under conditions of nutrient deprivation for 4 h. For the inhibition of autophagy, the cells were treated with 10 µM chloroquine (CQ; C6628, Sigma-Aldrich) in a complete medium for 4 h.

The cells were seeded in 10 cm dishes (2.0 × 10^6^ cells/dish) for immunoprecipitation, 60 mm dishes (5 × 10^5^ cells/dish) for immunoblotting, and 6-well plates (4 × 10^5^ cells/well) for immunofluorescence microscopy assay. After incubation for 24 h later, the cells were transfected with plasmid DNA or siRNA using Lipofectamine 2000 according to the manufacturer’s instructions (Invitrogen, Carlsbad, CA, USA). The samples were collected 48 h after transfection.

### 2.4. Immunoprecipitation (IP) and Immunoblotting

The cells were harvested through centrifugation and lysed by douncing 30 times in a lysis buffer composed of 1% NP-40, 2.6 mM KCl, 1.5 mM KH_2_PO_4_, 140 mM NaCl, 8 mM Na_2_HPO_4_-7H_2_O, and 1× protease inhibitor cocktail. The lysates were centrifuged for 15 min at 12,000 rpm. For IP, the cell lysates were incubated with FLAG or Myc antibodies overnight at 4 °C, and subsequently incubated with protein A or G sepharose for 3 h at 4 °C with gentle rocking. The samples were centrifuged and washed thrice with lysis buffer. The samples were added to the sample buffer and boiled for 5 min at 95 °C. For immunoblot analysis, the cells were incubated in a lysis buffer (20 mM Tris-HCl [pH 7.5], 150 mM NaCl, 1 mM Na_2_EDTA, 1 mM EGTA, 1% NP-40, 1% sodium deoxycholate, 2.5 mM sodium pyrophosphate, 1 mM β-glycerophosphate, 1 mM Na_3_VO_4_, and 1 µg/mL leupeptin with complete protease inhibitors) on ice for 15 min, followed by centrifugation at 13,000 rpm for 15 min at 4 °C. Proteins were extracted and measured using the Pierce BCA Protein Assay Kit (#23227, Thermo Fisher Scientific). Equal quantities of protein were loaded onto SDS-PAGE, transferred to PVDF membranes (Millipore, Billerica, MA, USA), and incubated with primary antibodies, followed by horseradish peroxidase-conjugated secondary antibodies. Band density was quantified using the ImageJ software version 1.54 K. The protein levels were adjusted to β-actin.

### 2.5. Mass Spectrometric (MS) Analysis

RhoBTB3 interaction partners were identified by mass spectrometry conducted at the Proteomics Core Facility at the National Cancer Center in South Korea [30]. The immunoprecipitated RhoBTB3 samples, as described above, were separated by SDS-PAGE. The gel was stained with the Pierce Silver Stain for Mass Spectrometry kit (#24600, Thermo Fisher Scientific) to visualize the protein bands according to the manufacturer’s instructions. After staining, the RhoBTB3 band was excised and subjected to in-gel tryptic digestion. The dried gel fragments were incubated in 30 µL of 25 mM sodium bicarbonate buffer (pH 8.8) with 50 ng of trypsin (Promega, Madison, WI, USA) and incubated overnight at 37 °C. The samples were purified using Zip-Tips C18 (EMD Millipore, Billerica, MA, USA) for desalting and then dissolved in 10 µL of 2% acetonitrile in 0.1% formic acid. The analysis was carried out using an LTQ XL linear ion trap mass spectrometer (Thermo Fisher Scientific). The spray voltage was set to +1.7 kV, with the capillary temperature maintained at 200 °C. The capillary voltage was adjusted to +20 V, the tube lens voltage to +100 V, and auxiliary gas at zero. Full scan analyses were conducted within the mass–charge ratio (m/z) range of 150–2000 using the linear ion trap mass spectrometer. Systematic MS/MS experiments were performed by varying the relative collision energy and evaluating the intensities of the fragment ions. Data processing was performed using the SEQUEST algorithm (Thermo Fisher Scientific, version v.27, rev. 11.), querying amino acid sequences in the SwissProt database (October 2018, https://ftp.uniprot.org/pub/databases/uniprot/current_release/knowledgebase/complete, accessed on 2 October 2024) and the International Protein Index (IPI) human database (October 2018, https://www.ebi.ac.uk/IPI, accessed on 2 October 2024). The SEQUEST search was performed with a parent ion tolerance of 1.2 Da and a fragment ion mass tolerance of 1.00 Da was applied, with methionine oxidation designated as a variable modification.

### 2.6. In Vivo Ubiquitination Assay

The ubiquitination of AMBRA1 in the cells was examined through a cell-based ubiquitination assay. Briefly, FLAG-RhoBTB3-, Myc-AMBRA1-, or HA-Ub-expressing cells were treated with MG132 for 4 h before harvesting. To detect proteins ubiquitinated with HA-conjugated ubiquitin under denaturing conditions, the cells were lysed by boiling for 10 min in Dulbecco’s Phosphate-Buffered Saline (DPBS; #LB 001–02, Welgene) containing 1% SDS and 5 mM N-ethylmaleimide (NEM; E3876, Sigma-Aldrich,). The lysates were then immunoprecipitated with anti-Myc antibody, followed by immunoblotting. Subsequently, 3 mg of whole cell lysates were precleared using protein A/G Sepharose beads (GE Healthcare) for 3 h and then incubated overnight at 4 °C with 3 mg of anti-Flag antibody (Sigma-Aldrich, F3165) for each sample. Finally, the immunocomplexes were captured on protein A/G-Sepharose beads, washed six times with lysis buffer, and either used for the in vivo ubiquitylation assay or eluted by boiling in SDS loading buffer and then subjected to SDS-PAGE.

### 2.7. Immunofluorescence Microscopy

Autophagic Flux Assay: The HeLa and A549 cells were seeded in 6-well plates on acid-washed glass coverslips. After 24 h, the cells were transfected with mRFP-EGFP-LC3 using Lipofectamine 2000 (Invitrogen) for 24 h. Following transfection, the cells were treated with 10 µM CQ for 4 h, then fixed with 4% paraformaldehyde for 30 min and washed five times with DPBS. Images were obtained using a confocal laser microscope (Zeiss; Carl Zeiss, Oberkochen, Germany), and merged images were processed using the ZEISS ZEN Microscopy Software 3.0 blue edition. For the quantification of autophagic cells, GFP-LC3 and mRFP-LC3 puncta were counted in triplicate samples.

### 2.8. Quantitative Reverse Transcription Polymerase Chain Reaction (RT-qPCR)

Total RNA was extracted using the RNeasy Mini Kit (QIAGEN, Valencia, CA, USA) according to the manufacturer’s instructions. cDNA was synthesized from total RNA using the Superscript II First-Strand Synthesis kit and analyzed via real-time PCR using the Light 480 SYBR Green Supermix kit (Roche, Mannheim, Germany) with the following primers: human RhoBTB3: 5′-GTGTTGTGCGSTGSCG-3′ and 5′-TCGGTGGGTGACTCT-3′; human AMBRA1: 5′-CTGGTGTATCTTTAGGTGGTG-3′ and 5′-GTGCTGGTGGGATG TTG-3′; human β-actin: 5′-CACCATTGGCAATGAGCGGTTC-3′ and 5′-AGGTCTTTGCGGATGTCCACGT-3′. The gene expression data were normalized to that of β-actin.

### 2.9. Statistical Analyses

For all the experiments presented, the sample size (n) is specified in the legends of the figures. Densitometric analysis was conducted using the ImageJ software, using the average values from multiple experiments (as noted) normalized to a control ratio, which was arbitrarily set to 1.00. Each data point represents the mean ± standard error of the mean (SEM) or standard deviation (SD) from three independent experiments unless stated otherwise. The comparisons of Western blot intensity between control and sample groups were made using the same blot. Statistical significance was determined using an unpaired *t*-test.

## 3. Results

### 3.1. RhoBTB3 Regulates Autophagy

**Figure 1:**
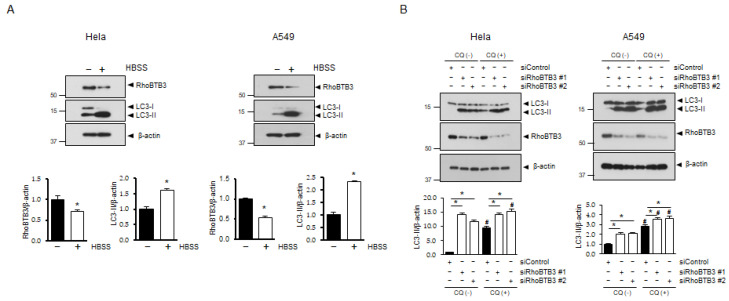

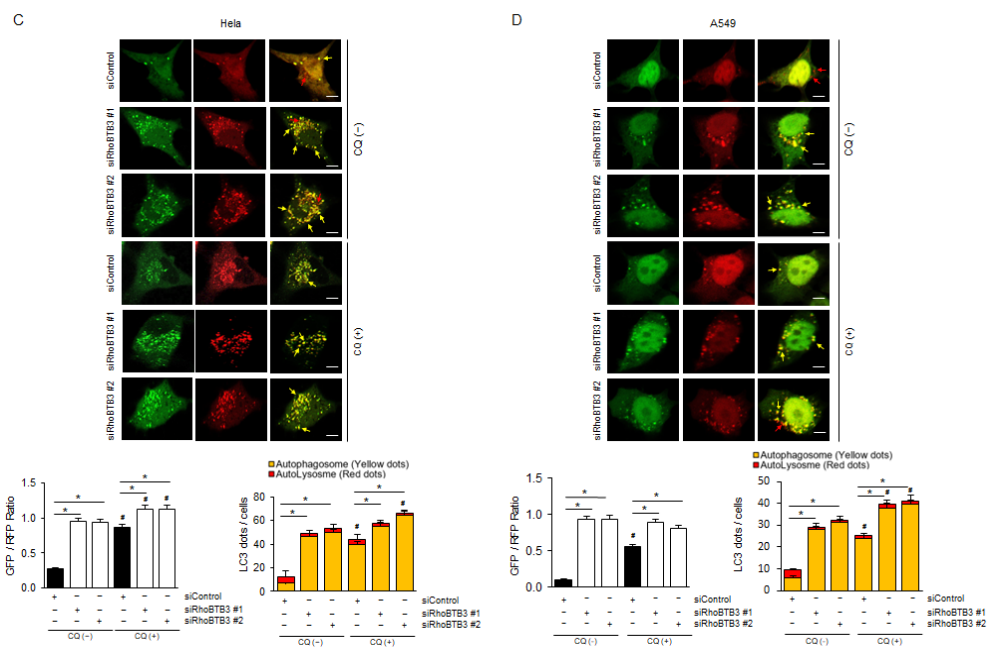
Effect of RhoBTB3 on autophagy. (**A**) The HeLa and A549 cells were treated with HBSS (+) or without HBSS (−), indicating treatment in complete medium, for 4 h. RhoBTB3, LC3, and β-actin were analyzed by immunoblotting. The quantification of the RhoBTB3 and LC3II bands is shown in the lower panel. *n* = 3, data are shown as mean ± SD and * *p* < 0.01. (**B**) The HeLa and A549 cells were transfected with siControl or siRhoBTB3. All the samples were analyzed 48 h after transfection, either untreated or treated with 10 µM chloroquine (CQ) for 4 h. The lysates were subjected to immunoblotting using the indicated antibodies. The quantification of the LC3II bands is shown in the lower panel. *n* = 3, data are presented as mean ± SD, with * *p* < 0.05 indicating significance, while # *p* < 0.05 compares the CQ (−) group to the CQ (+) group. (**C**,**D**) The representative confocal microscopy images of the GFP-RFP-LC3 assay in the RhoBTB3-knockdown HeLa and A549 cells after treatment with 10 µM CQ for 4 h. The arrows indicate autophagosomes (yellow dots) and autolysosomes (red dots). Scale bar: 10 μm. *n* = 3, data are presented as mean ± SD, with * *p* < 0.05 indicating significance for autophagosomes, not autolysosomes, while # *p* < 0.05 compares the CQ (−) group to the CQ (+) group for autophagosomes, not autolysosomes.

**Figure 2:**
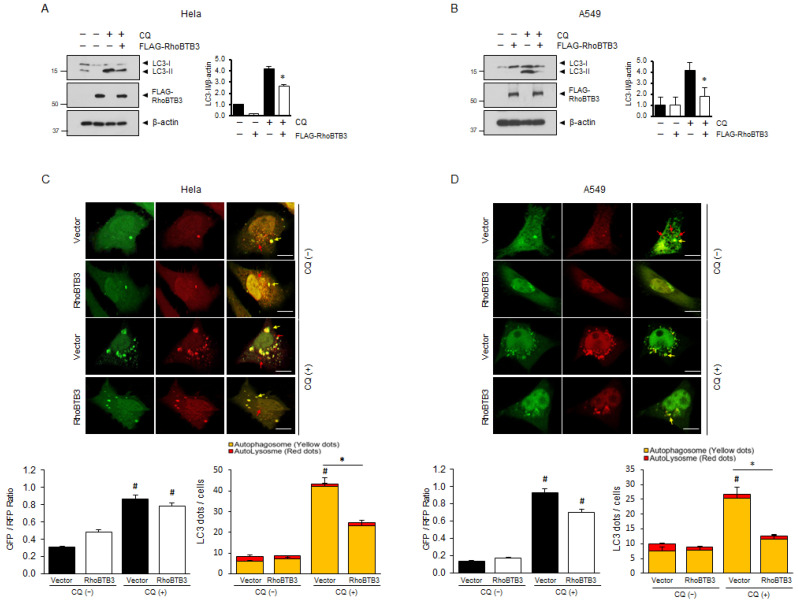
Effect of RhoBTB3 overexpression on the chloroquine-induced accumulation of autophagosomes. (**A**,**B**) The HeLa and A549 cells were transfected with Vector or FLAG-RhoBTB3. All the samples were analyzed 48 h after transfection, either untreated or treated with 10 µM chloroquine (CQ) for 4 h. The lysates were subjected to immunoblotting using the indicated antibodies. The quantification of the LC3II bands is shown in the lower panel. *n* = 3, data are shown as mean ± SD and * *p* < 0.05. (**C**,**D**) The representative confocal microscopy images of the GFP-RFP-LC3 assay in the RhoBTB3-overexpressing or vector control HeLa and A549 cells after treatment with 10 µM CQ for 4 h. The arrows indicate autophagosomes (yellow dots) and autolysosomes (red dots). Scale bar: 10 μm. *n* = 3, data are presented as mean ± SD, with * *p* < 0.05 indicating significance for autophagosomes, not autolysosomes, while # *p* < 0.05 compares the CQ (−) group to the CQ (+) group for autophagosomes, not autolysosomes.

To investigate the relationship between RhoBTB3 and autophagy, we used a classical autophagic stimulus (HBSS) and examined changes in RhoBTB3 expression. As shown in Figure 1A, the HBSS treatment caused a significant increase in microtubule-associated proteins 1A/1B light chain 3A (LC3) lipidation and a decrease in RhoBTB3 expression in the treated cells compared with the cells in the control group. To verify the role of RhoBTB3 in autophagy, the cells were treated with RhoBTB3 siRNA and chloroquine (CQ), and the autophagic process was studied by analyzing the conversion of LC3I to LC3II. After treatment with CQ, a significant increase in LC3II lipidation was observed in the RhoBTB3-downregulated cells compared with that in the control cells. Moreover, as shown in Figure 1B, LC3-II lipidation was significantly increased in the RhoBTB3-downregulated cells even in the absence of the CQ treatment. To determine whether the deletion of RhoBTB3 affects autophagosome formation or turnover, we used a GFP-RFP tandem fluorescent-tagged LC3 (GFP-RFP-LC3) and examined autophagosome dynamics. In the RhoBTB3 knockdown cells where GFP-RFP-LC3 was overexpressed, we observed the formation of double GFP/RFP-positive puncta (yellow), which represent early autophagosomes, as depicted in Figure 1C and D. These findings support the hypothesis that the loss of RhoBTB3 can activate the autophagic pathway, leading to increased autophagosome formation. Subsequently, we investigated the potential inhibitory effect of RhoBTB3 overexpression on autophagy. After treating the cells with chloroquine (CQ) for 4 h, we observed a significant reduction in the conversion of LC3II in the cells overexpressing RhoBTB3 compared to the vector control (Figure 2A,B). Furthermore, to assess autophagic activity, we introduced GFP-RFP-LC3 into the RhoBTB3-overexpressing cells. Notably, we observed a reduction in the formation of GFP-RFP-positive puncta (Figure 2C,D). These findings support the idea that RhoBTB3 inhibits the formation of autophagosomes. Collectively, these data demonstrate that RhoBTB3 deficiency induces autophagy, whereas RhoBTB3 overexpression inhibits autophagosome formation. Therefore, our data suggest that RhoBTB3 plays an important role in autophagosome formation.

### 3.2. AMBRA1 Is a Substrate of RhoBTB3

**Figure 3:**
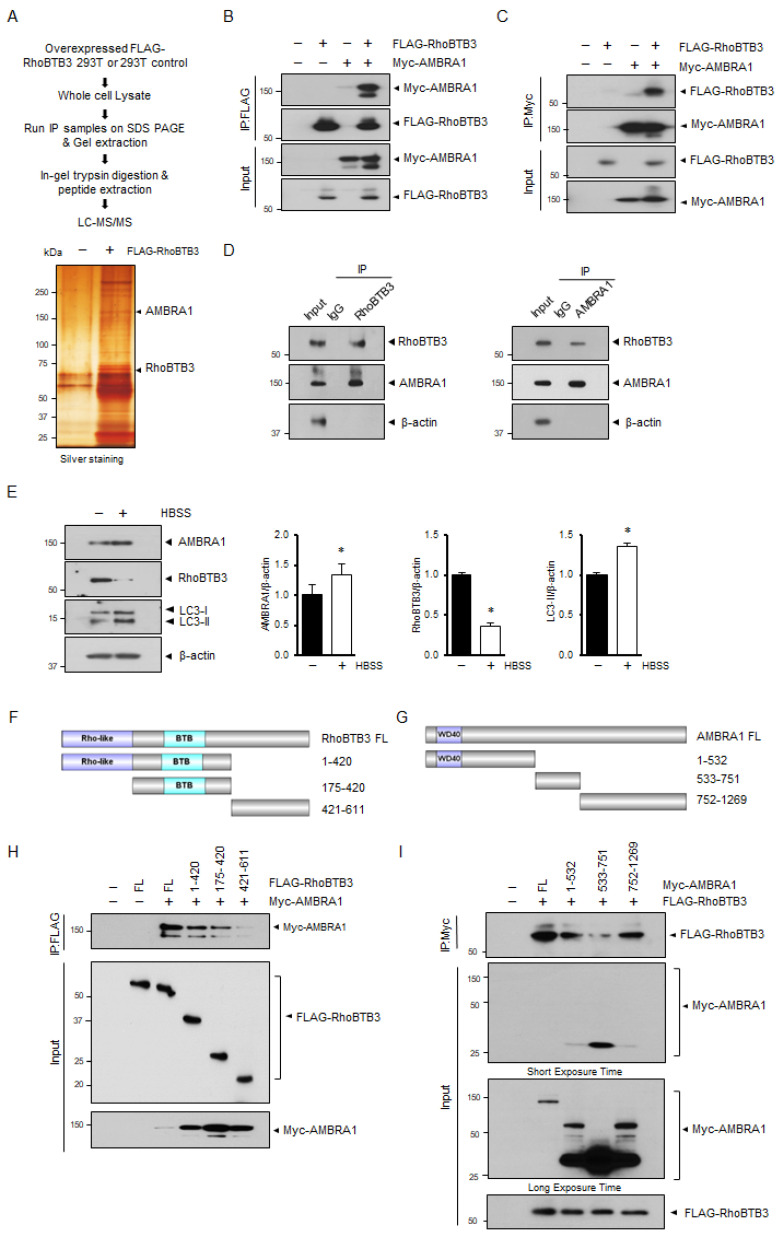
RhoBTB3 interacts with AMBRA1. (**A**) Diagram depicting the immunoprecipitation-mass spectrometry (IP-MS) approach used to identify RhoBTB3 interacting proteins. Immunoprecipitates from the 293T cells stably expressing FLAG-RhoBTB3 or the empty vector (−) were resolved by SDS-PAGE and stained with silver nitrate for protein visualization. Immunoprecipitation was conducted using anti-FLAG antibody or IgG, and the samples were analyzed via LC/MS. (**B**,**C**) FLAG-RhoBTB3, Myc-AMBRA1, and empty vector (−) were co-transfected into the 293T cells, followed by IP with antibodies against FLAG and Myc. (**D**) RhoBTB3 interacts with endogenous AMBRA1, followed by IP with antibodies against FLAG and AMBRA1. (**E**) The immunoblotting analysis of AMBRA1, RhoBTB3, and LC3 in the 293T cells treated with HBSS (+) or without HBSS (−), indicating treatment in complete medium, for 4 h. The ImageJ densitometry analysis of independent experiments. *n* = 3, data are shown as mean ± SD and * *p* < 0.05. (**F**,**G**) RhoBTB3 and AMBRA1 constructs are illustrated. (**H**) The 293T cells were co-transfected with a vector control (−) and a vector encoding AMBRA1 together with FLAG- RhoBTB3 FL (full length) or its domain mutants. The protein extracts were immunoprecipitated using an anti-FLAG antibody. The relative levels of co-immunoprecipitated Myc-AMBRA1 and FLAG-RhoBTB3 FL, as well as their deletion mutants, were analyzed. (**I**) The 293T cells were co-transfected with a vector control (−) and a vector encoding FLAG-RhoBTB3 together with Myc-AMBRA1 FL or its domain mutants. The protein extracts were immunoprecipitated using an anti-FLAG antibody. The relative levels of co-immunoprecipitated RhoBTB3 and Myc-AMBRA1 FL or their deletion mutants were consistent.

RhoBTB3 has also been identified as a component of the Cullin-dependent E3 ubiquitin ligase complex, which plays a role in the degradation of its interacting partners [25,31]. To identify specific autophagic substrates of RhoBTB3, we utilized immunoprecipitation (IP) coupled with mass spectrometry (MS), as outlined in Figure 3A. The cells were transfected with either FLAG-RhoBTB3 or a control vector. The cell lysates underwent IP using an anti-FLAG antibody conjugated to agarose beads, and the protein samples were separated via SDS-PAGE, followed by visualization using silver staining (Figure 3A). Notably, the FLAG-RhoBTB3-transfected cell lysates exhibited more protein bands than the lysates of the cells transfected with the empty vector. These bands were excised from the gel, and the interacting proteins were analyzed through MS, which revealed peptide sequences of various proteins. Among these proteins, AMBRA1 had the strongest known link to the autophagy pathway. To confirm the interaction between the two proteins, we conducted reciprocal IPs using FLAG-RhoBTB3 and Myc-AMBRA1 (Figure 3B,C). Additionally, we validated the interaction between endogenous RhoBTB3 and AMBRA1 through IP (Figure 3D). To further explore the relationship between RhoBTB3 and AMBRA1 in autophagy in the HBSS-treated cells, we assessed the expression of RhoBTB3 and AMBRA1 and the conversion of LC3. Our findings revealed a decline in RhoBTB3 expression, whereas the expression of AMBRA1 and conversion of LC3 increased in response to HBSS treatment (Figure 3E). To determine the interacting domains of RhoBTB3 and AMBRA1, we employed deletion constructs. The RhoBTB3 deletion constructs included the following: (1) the N-terminal region (1–420) containing a Rho GTPase and BTB domain, (2) the central region (176–419) containing a BTB domain, and (3) the C-terminal region (421–611) (Figure 3F). Similarly, the AMBRA1 deletion constructs included the following: (1) the N-terminal region (1–532) containing a WD40 domain, (2) the central region (533–751), and (3) the C-terminal region (751–1269) (Figure 3G). Our results demonstrated that the full-length RhoBTB3 protein and its Rho GTPase and BTB domain regions interacted with AMBRA1, whereas the C-terminal region of RhoBTB3 failed to bind AMBRA1 (Figure 3H). Consistent with these findings, when mapping the AMBRA1 region responsible for RhoBTB3 binding, we observed that the N- and C-terminal regions of AMBRA1 interacted with RhoBTB3, whereas the central region of AMBRA1 weakly bound to RhoBTB3 (Figure 3I).

### 3.3. RhoBTB3 Regulates AMBRA1 Stability

**Figure 4:**
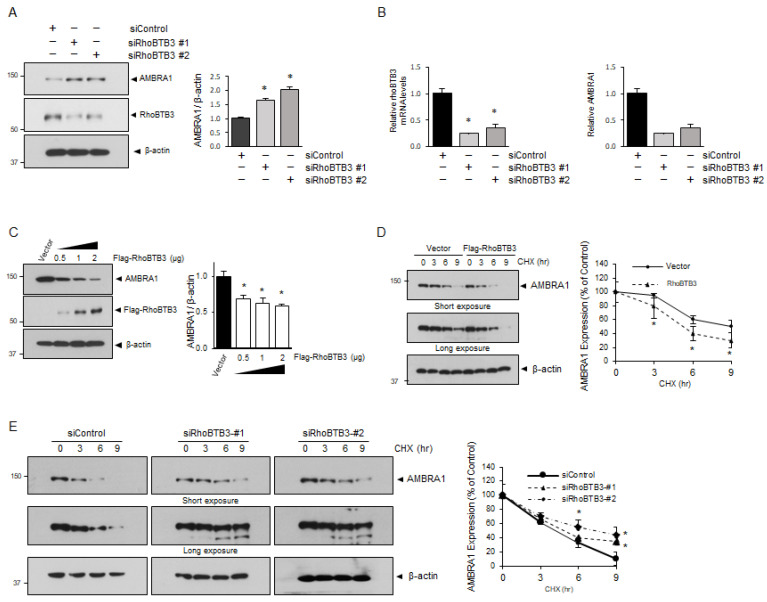
RhoBTB3 regulates AMBRA1 stability. (**A**) RhoBTB3 depletion elevates AMBRA1 expression. The 293T cells were transfected with siControl or siRhoBTB3. The lysates were analyzed using immunoblotting, and the quantification of the AMBRA1 and RhoBTB3 bands is shown in the right panel. *n* = 3, data are shown as mean ± SD and * *p* < 0.05. (**B**) RhoBTB3 deficiency does not change AMBRA1 mRNA levels. The siControl and siRhoBTB3 cells were harvested, and the mRNA levels of RhoBTB3 and AMBRA1 were analyzed using qPCR. The mRNA levels of AMBRA1 were normalized to those of actin. *n* = 3, data are shown as mean ± SD and * *p* < 0.01. (**C**) The 293T cells were transfected with 0.5, 1, or 2 µg of FLAG-RhoBTB3 plasmid DNA. The cell lysates were subjected to immunoblotting, and the quantification of the AMBRA1 band is shown in the right panel. *n* = 3, data are shown as mean ± SD and * *p* < 0.05. (**D**) RhoBTB3 overexpression decreases endogenous AMBRA1 stability. The cells were transfected with the AMBRA1 for 48 h and treated with CHX at the indicated time points. ImageJ densitometry analysis was performed for independent experiments. *n* = 3, data are shown as mean ± SD and * *p* < 0.05 (**E**) RhoBTB3 deficiency prevents AMBRA1 degradation. The 293T cells were transfected with either siControl or siRhoBTB3, and then CHX was added at specified time points. The graph represents the values derived from the densitometry analysis. The percentage of remaining AMBRA1 protein after the addition of CHX is represented in the plot. *n* = 3, data are shown as mean ± SD and * *p* < 0.05.

To investigate the role of RhoBTB3 in AMBRA1 stability, we performed immunoblotting experiments following the RhoBTB3 knockdown and overexpression. The results showed that the RhoBTB3 knockdown increased AMBRA1 protein levels, whereas the overexpression of 0.5, 1, or 2 µg of RhoBTB3 DNA led to a decrease in AMBRA1 protein levels in a RhoBTB3 expression-dependent manner (Figure 4A,C). In addition, we assessed the AMBRA1 mRNA levels in the RhoBTB3 knockdown cells and found no significant effect, suggesting that RhoBTB3 regulates AMBRA1 protein levels but not its mRNA levels (Figure 4B). To examine whether RhoBTB3 influences AMBRA1 stability, we analyzed the AMBRA1 protein levels in the RhoBTB3 knockdown and overexpressed cells after treatment with the protein synthesis inhibitor cycloheximide (CHX). The results showed that AMBRA1 degradation was significantly increased in the RhoBTB3-overexpressing cells (Figure 4D) and significantly decreased in the RhoBTB3 knockdown cells (Figure 4E). These findings support the hypothesis that RhoBTB3 plays a role in regulating AMBRA1 stability.

### 3.4. RhoBTB3 Induces the Ubiquitination and Proteasome-Mediated Degradation of AMBRA1

**Figure 5:**
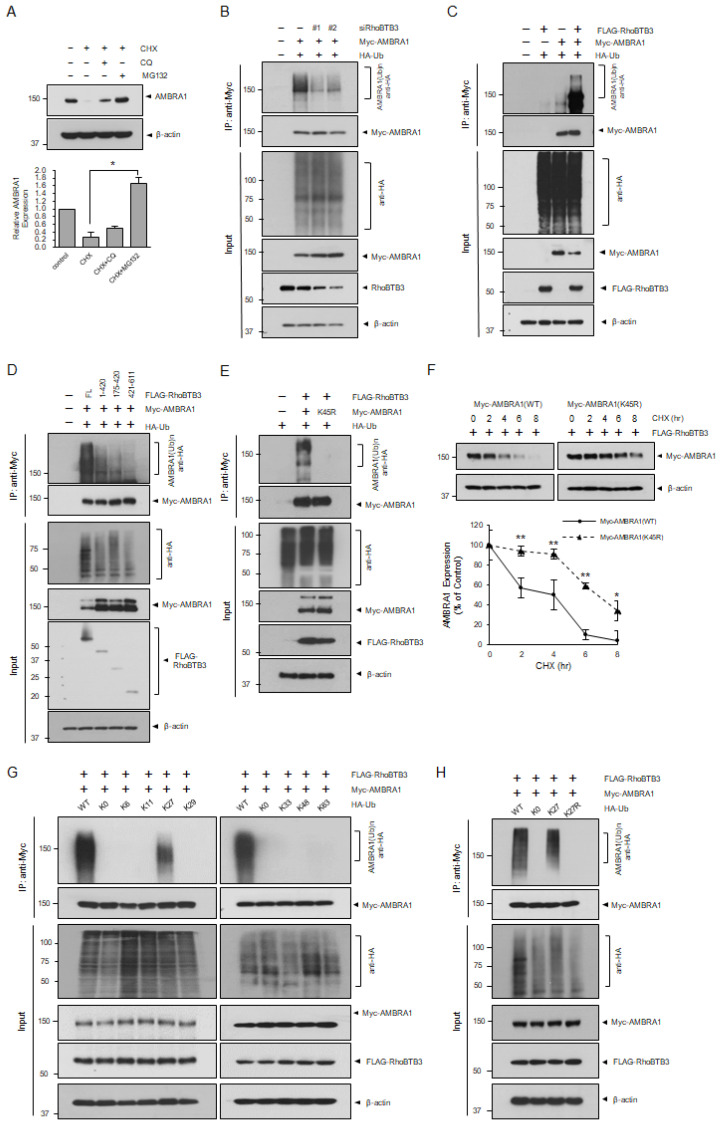
RhoBTB3 regulates AMBRA1 degradation via the ubiquitin–proteasome pathway. (**A**) AMBRA1 is degraded via the proteasomal degradation pathway. The cells were treated with the indicated reagents (20 μg/mL CHX, 20 μM CQ, and 10 μM MG132) for 6 h. *n* = 3, data are shown as mean ± SD and * *p* < 0.05. (**B**) The immunoprecipitation analysis of AMBRA1 ubiquitination in the cells transfected with the indicated constructs. (−) indicates sicontrol or empty vector. (**C**) The immunoprecipitation analysis of AMBRA1 ubiquitination in the cells transfected with the indicated constructs, either vector control (−) or RhoBTB3. (**D**) The RhoBTB3 mutant was co-transfected with AMBRA1 and HA-tagged ubiquitin (HA-Ub) into cells for 48 h. (−) indicates the empty vector. The cells were harvested after pretreatment with 10 μM MG132 for 6 h, followed by immunoprecipitation (IP) with an antibody against Myc-AMBRA1. (**E**) The AMBRA1 mutant was co-transfected with RhoBTB3 with HA-Ub into the cells for 48 h. (−) indicates an empty vector. The cells were harvested after pretreatment with 10 μM MG132 for 6 h, followed by IP with antibody against Myc-AMBRA1. (**F**) The AMBRA1-silenced cells were transfected with WT-AMBRA1 or K45R-AMBRA1. The cells were incubated for the indicated times with 20 μg/mL CHX. The cells were then harvested and used for immunoblotting with the indicated antibodies. The ImageJ densitometry analysis of independent experiments. *n* = 3, data are shown as mean ± SD and * *p* < 0.05. (**G**,**H**) Ubiquitination reactions catalyzed by FLAG-RhoBTB3 and Myc-AMBRA1 with the indicated constructs and ubiquitin or the indicated ubiquitin variants with lysine-to-arginine substitutions, followed by IP with an antibody against Myc-AMBRA1. The error bars indicate the mean ± SD, * *p* < 0.05, ** *p* < 0.01.

Protein degradation is primarily regulated by proteasomal and lysosomal pathways, which maintain homeostasis [32,33]. We investigated how RhoBTB3 regulates AMBRA1 stability. Our findings indicated that, under normal conditions, MG132, a proteasome inhibitor, blocked AMBRA1 degradation more effectively than CQ, a lysosome inhibitor, suggesting AMBRA1 is primarily degraded via the proteasome pathway (Figure 5A). We predicted that RhoBTB3 controls AMBRA1 stability through ubiquitination and examined this hypothesis by transfecting cells with siRNA against RhoBTB3, Myc-AMBRA1, and HA-tagged Ub (HA-Ub). The results indicated a reduction in the levels of ubiquitinated AMBRA1 in the cells with RhoBTB3 knockdown (Figure 5B). To further confirm this, we transfected cells with FLAG-RhoBTB3, Myc-AMBRA1, and HA-Ub and found that RhoBTB3 mediates AMBRA1 ubiquitination (Figure 5C). As shown in Figure 2D, we confirmed that the Rho-like and BTB domain regions of RhoBTB3 interact with AMBRA1. Therefore, we conducted a ubiquitination assay utilizing different domains of RhoBTB3 to determine where ubiquitination occurs during the binding of AMBRA1. Consequently, when we used the Rho-like and BTB domain regions of RhoBTB3 with HA-Ub, the ubiquitination of AMBRA1 was observed (Figure 5D). The AMBRA1 N-terminal region containing lysine 45 (K45) is ubiquitinated by RNF2 [34]. We transfected AMBRA1 wild-type (WT) or AMBRA1 mutants with Ub into cells. To determine whether RhoBTB3 ubiquitinates K45, a ubiquitination assay was performed using AMBRA1 WT and AMBRA1 (K45R) mutants. We found that AMBRA1 (K45R) was significantly less ubiquitinated than AMBRA1 (Figure 5E). In the presence of RhoBTB3, the degradation of the AMBRA1 protein was less in AMBRA1 (K45R) compared to AMBRA1 WT (Figure 5F). Accordingly, our data suggest that AMBRA1 degradation is due to K45 ubiquitination by RhoBTB3. Ub has seven lysine residues—K6, K11, K27, K29, K33, K48, and K63—through which ubiquitin chains are formed [35]. To characterize the Ub linkage preference of AMBRA1, we performed ubiquitination assays using K-only Ub linkage, and all seven lysine residues altered HA-Ub K0. These results showed that K27-linked ubiquitination was the most common linkage type used by RhoBTB3 to ubiquitinate AMBRA1 (Figure 5G). To confirm that AMBRA1 is ubiquitinated by RhoBTB3 via K27 linkage, we performed a ubiquitination assay using HA-Ub K27 and HA-Ub K27R mutants in the presence of RhoBTB3. The results showed that the ubiquitination level of AMBRA1 was significantly decreased in HA-Ub K27R compared to that in HA-Ub K27 (Figure 5H). These data suggested that Ub K27 is important for AMBRA1 ubiquitination in the presence of RhoBTB3. Collectively, our data demonstrated that the RhoBTB3-mediated ubiquitination of the AMBRA1 K45 residue occurred via the Ub K27 linkage.

### 3.5. RhoBTB3-Induced Autophagy Inhibition Is Dependent on AMBRA1

**Figure 6:**
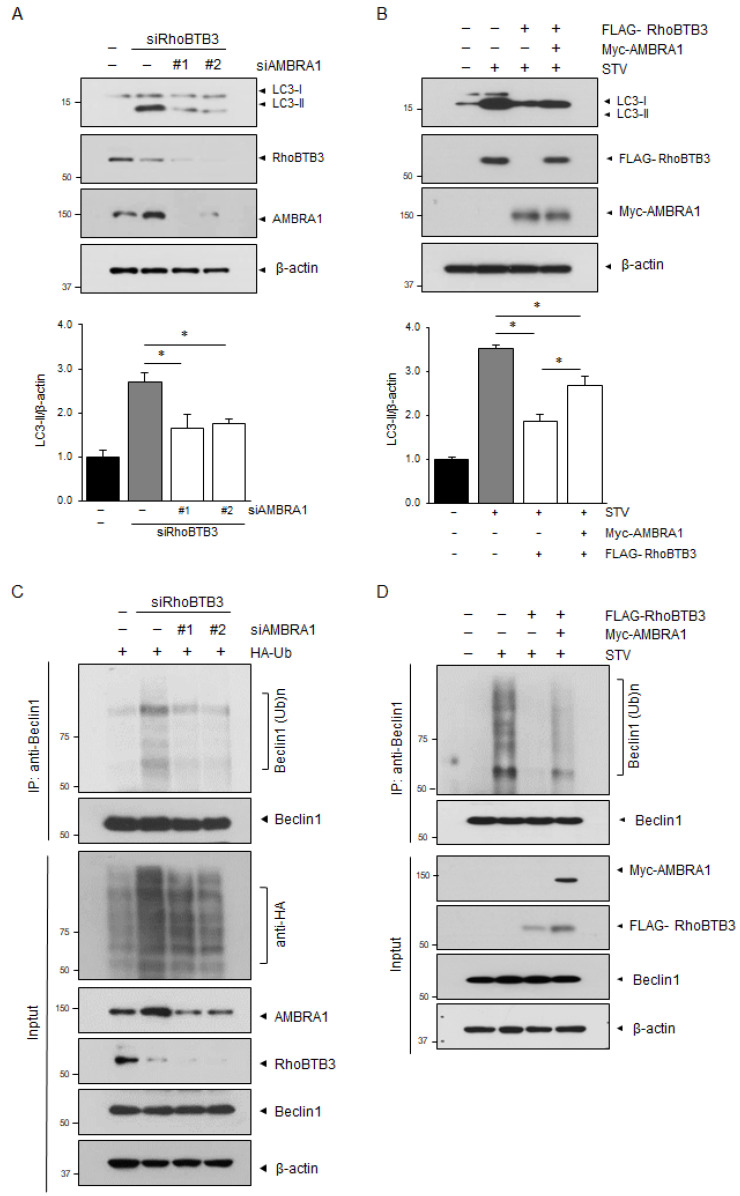
RhoBTB3 regulates autophagy-related functions via AMBRA1 degradation RhoBTB3 regulates autophagy-related functions via AMBRA1 degradation. (**A**) The 293T cells were transfected with siControl, siRNA targeting RhoBTB3, or siRNA targeting AMBRA1, as indicated. (**B**) The 293T cells were transfected with a FLAG-RhoBTB3 encoding vector in combination with plasmids encoding Myc-AMBRA1, as indicated, and then subjected to 6 h of starvation (STV). For both (**A**,**B**), LC3, RhoBTB3, and AMBRA1 protein levels were analyzed by immunoblotting using anti-LC3, anti-RhoBTB3, and anti-AMBRA1 antibodies, respectively. β-actin was used as a loading control. The data are presented as mean ± SD, with *n* = 3. Statistical significance was determined with * *p* < 0.05. (**C**) The 293T cells were transfected with siControl, siRNA targeting RhoBTB3, or siRNA targeting AMBRA1, as indicated, and then harvested for immunoprecipitation (IP) using an antibody against Beclin1. The immunoprecipitates and cell lysates were subsequently immunoblotted with the indicated antibodies. (**D**) The 293T cells were transfected with a FLAG-RhoBTB3 encoding vector in combination with plasmids encoding Myc-AMBRA1, as indicated, and then subjected to 6 h of starvation (STV). The immunoprecipitates and cell lysates were subsequently immunoblotted with the indicated antibodies.

We analyzed the siRNAs that target AMBRA1 and found that two of the siRNAs were effective in silencing AMBRA1 expression. These two AMBRA1-specific siRNAs were able to block RhoBTB3 knockdown-induced autophagy, as demonstrated by their ability to prevent RhoBTB3 knockdown from causing an accumulation of LC3B-II (Figure 6A). Consistently, the overexpression of AMBRA1 also rescued the effect of RhoBTB3 overexpression on the inhibition of LC3B-II accumulation (Figure 6B). As previously mentioned, AMBRA1 directly binds to and mediates the ubiquitylation of Beclin1, leading to the formation of autophagosomes [36,37,38]. Therefore, we tested whether RhoBTB3 regulates Beclin1 ubiquitination by controlling AMBRA1 stability. We further investigated Beclin1 ubiquitination in RhoBTB3 knockdown or overexpressing cells. RhoBTB3 knockdown significantly increased the ubiquitination of Beclin1 upon autophagy induction, while the knockdown of both RhoBTB3 and AMBRA1 decreased Beclin1 ubiquitination (Figure 6C). In contrast, RhoBTB3 overexpression drastically decreased Beclin1 ubiquitination; however, the overexpression of both RhoBTB3 and AMBRA1 rescued Beclin1 ubiquitination compared to RhoBTB3 overexpression alone. (Figure 6D). The results showed that RhoBTB3 inhibited autophagy by suppressing Beclin1 ubiquitination through the destabilization of AMBRA1. These findings suggest that RhoBTB3 regulates the interaction involving Beclin1 by controlling AMBRA1 stability, thereby affecting autophagosome formation.

## 4. Discussion

Autophagy is a crucial mechanism for the maintenance of cellular and organismal homeostasis [2]. It is a highly conserved catabolic process in which cytoplasmic components are transported to lysosomes for degradation via autophagosomes [39,40]. However, the relationship between RhoBTB3 and autophagy has not been explored. This study provides evidence that RhoBTB3 negatively regulates autophagy through its interaction with AMBRA1, leading to proteasomal degradation by the ubiquitination of AMBRA1. It inhibits Beclin1 ubiquitination, consequently suppressing autophagy.

The process of autophagic flux includes the formation of autophagosomes, their fusion with lysosomes, and the subsequent degradation of proteins and organelles. In the recent studies, E3 ligases, such as Cullins, TRIM23, and RNF2, were recently found to regulate autophagosome formation in the autophagy process [32,39,40]. In this study, we identified RhoBTB3, an E3 adaptor protein in the RhoBTB subfamily, as a negative regulator of autophagosome formation. Notably, the basal level of LC3II lipidation is significantly increased upon RhoBTB3 loss. While LC3-II levels rose under siRhoBTB3 conditions for both siRNAs; the lack of discernible differences between CQ- and CQ+ conditions suggests that RhoBTB3 may inhibit the fusion of autophagosomes with lysosomes. This implies that although autophagosomes are being formed, degradation is not occurring, thereby blocking the autophagic flux.

RhoBTB3 interacts with various protein substrates, including MUF1/LRRC41, cyclin E, HIF1A, and Rab9 [25,27,28,41]. Through an IP-MS analysis, we found that RhoBTB3 mediates the interaction with AMBRA1 among several autophagy-related proteins. AMBRA1, a positive regulator of autophagy, plays an important role in autophagosome formation [41,42]. RhoBTB proteins comprise the domain architecture, including N-terminal regions containing the Rho domain, central regions containing the BTB domain, and C-terminal regions [26,43]. Rab9 has been shown to interact with the C-terminal region of RhoBTB3 [24]. Cyclin E interacts with both the N- and C-terminal regions of RhoBTB3 [29]. Similarly, the binding region of a protein that interacts with RhoBTB3 depends on the specific binding partner. Our results show that AMBRA1 interacts with the N-terminal regions containing the BTB domain of RhoBTB3 but not with the C-terminal regions. AMBRA1 contains a WD40 domain [44]. WD40-domain proteins facilitate the assembly of multi-protein complexes and generally serve as a stable scaffold for protein–protein interactions [45]. Beclin1 binds to the central region of AMBRA1, and DLC1 binds to the C-terminal region of AMBRA1 [17]. ULK1 interacts with the N-terminal and C-terminal regions of AMBRA1 [20]. Similarly, we found that the N- and C-terminal regions of AMBRA1 were sufficient to interact with RhoBTB3. Notably, the 533–751 fragment of AMBRA1 was found to be more abundant than the other fragments. The decreased interaction with RhoBTB3 may contribute to the prevention of the degradation of this fragment.

AMBRA1 function is regulated by post-translational modifications, including phosphorylation and ubiquitination, which play an important role in autophagy induction and progression [20,46]. AMBRA1 is regulated by the phosphorylation of the two kinases, mTOR and ULK1. mTORC1 interacts with AMBRA1 and it can phosphorylate AMBRA1, thereby regulating autophagy [20]. ULK1 also phosphorylates AMBRA1 and promotes the dissociation of the AMBRA1–Beclin1 complex, thereby regulating the nucleation process and autophagosome biogenesis [9,12,20]. Ubiquitination is another crucial post-translational modification that regulates the degradation of various proteins related to autophagy. Recently, it has been reported that RNF2, acting as an E3 ligase, ubiquitinates AMBRA1 and induces its degradation, consequently inhibiting autophagy in the presence of the FAM21-containing WASH complex [34]. The DDB1-Cullin4 E3 Ub ligase complex has also been shown to regulate AMBRA1 stability through ubiquitination, thereby inhibiting autophagy [37]. In this study, we identified RhoBTB3 as another novel factor necessary for the ubiquitination and destabilization of AMBRA1, ultimately leading to the inhibition of autophagy.

Ubiquitination regulates protein degradation through the formation of complex Ub chains linked by the interaction of various lysine residues (K6, K11, K27, K29, K33, K48, or K63) [47,48]. Among these, K48-linked Ub chains are typically associated with targeting proteins for degradation by the proteasome, a cellular complex responsible for breaking down and recycling proteins [49]. Recently, RNF2 has been observed ubiquitinating AMBRA1 at K45 with K48-linked Ub chains [34]. This modification promotes the degradation of AMBRA1 by the proteasome, resulting in reduced autophagy. In this study, we have identified the role of RhoBTB3 in destabilizing AMBRA1 at K45 through K27-linked ubiquitination, which is in contrast to RNF2′s use of K48-linked Ub chains on AMBRA1. K27-linked Ub chains have been implicated in various cellular processes, such as the regulation of protein–protein interactions, DNA repair, and immune response [50,51,52]. Unlike K48-linked chains, K27-linked chains have not been as extensively studied in the context of protein degradation. However, recently, the K27 Ub chain has been reported to regulate autophagy, inhibiting it through the LATS1-induced K27 ubiquitination of Beclin1 or facilitating autophagic degradation through the March2-mediated K27-linked polyubiquitination of IKKε [53,54]

In this regard, RhoBTB3 and RNF2 ubiquitinate AMBRA1 at the same position, K43, but there is a difference in the K48 and K27 Ub chain for this modification. The reason for the distinct use of K48 and K27 may be due to specific functional requirements to regulate the diverse cellular processes of the targeted proteins. Therefore, further research is needed to understand how this selection is regulated by specific cellular contexts and conditions.

In conclusion, our study identified a novel role for RhoBTB3 as a negative regulator of autophagy. The results showed that RhoBTB3 mediated K27 ubiquitination and the degradation of AMBRA1, leading to autophagy inhibition. The loss of RhoBTB3 negatively impacts cellular processes by enhancing AMBRA1 stability. Studies on the mechanisms of various human pathologies caused by autophagy dysfunction are steadily increasing. Based on our results, we propose that RhoBTB3 may be a potential therapeutic target for the modulation of AMBRA1 in autophagy-related diseases.

## Data Availability

Data are contained within the article.
